# Research Note: Effects of high barn temperature on group-level dispersion and individual activity in broiler chickens

**DOI:** 10.1016/j.psj.2024.103901

**Published:** 2024-05-24

**Authors:** Pascal Duenk, Esther D. Ellen, Ingrid C. de Jong, Malou van der Sluis

**Affiliations:** ⁎ Animal Breeding and Genomics, Wageningen University & Research, Wageningen, AH 6700, the Netherlands; † Animal Health and Welfare, Wageningen University & Research, Wageningen, AH 6700, the Netherlands

**Keywords:** heat stress, behavior, activity, dispersion, broiler

## Abstract

Heat stress in broilers is a pressing issue in the changing climate. Data on broiler behavior might be useful for early detection of heat stress and subsequent intervention, and may provide potential indicators for heat tolerance that can be used in broiler breeding programs. Here, we used bird location data collected in a previous study during which broilers were inadvertently exposed to high ambient temperatures due to a local heat wave. We examined whether broiler behavior changed with increasing ambient temperatures, focusing on group-level dispersion behavior and individual-level locomotor activity. We observed that birds moved closer together with increasing temperatures up to 9 °C above the desired level, and remained in similar proximity or moved further apart at temperatures above that threshold. The activity level decreased or remained stable with increasing temperature during most parts of the day, but increased at the end of the day. Possibly, the birds exhibited compensatory behavior (such as drinking and eating) during the periods when the barn cooled down after a hot day, but that could not be confirmed as no behavioral observations were available. The difference in activity levels between individuals accounted for 8.4% of the total variation, suggesting that activity might be an interesting indicator trait for heat tolerance in broiler chickens. Overall, the results of this study can inform the development of behavior-based 1) early-warning systems for heat stress and 2) heat tolerance indicators, although data on behaviors that are more specific to heat stress are probably required.

## INTRODUCTION

Heat waves are expected to become more frequent, longer-lasting, and more intense in the future due to climate change. During a heat wave, the indoor temperature in broiler barns can reach high levels, increasing the chance that birds experience heat stress, that is, they have difficulty in balancing body heat production and heat loss (Nascimento et al. 2017). Heat stress can cause a cascade of physiological responses, which in turn negatively affect production efficiency, health, welfare, and mortality (Saeed et al. 2019; Wasti et al. 2020; Oluwagbenga and Fraley 2023). Hence, there is a need to mitigate heat stress in broilers to improve the overall sustainability of broiler production.

There are various management interventions that can mitigate heat stress, such as additional ventilation, spraying of water, adjusting feeding strategies or using feed additives (Saeed et al. 2019; Wasti et al. 2020). However, such interventions may not always be sufficient and are often only implemented when heat stress already has a negative impact on the animals. For interventions to be effective and to prevent negative effects on broiler welfare, farmers need to be able to detect heat stress at a very early stage. Moreover, to allow breeding for improved heat tolerance, individual-level indicators of heat stress are of great importance.

Animals typically respond to environmental stressors through changing their behavior, which suggests that alterations in behavior might be the first visible indicator of thermal discomfort. Previous research has indeed suggested that environmental temperature is correlated to group-level behaviors such as clustering and general unrest (Pereira et al. 2020; Del Valle et al. 2021), and the frequencies of individual-level behaviors such as drinking, eating, panting, and roosting (Li et al. 2015; Cartoni Mancinelli et al., 2023). Data on these behaviors can be informative for both early detection of heat stress at the group level, and indicators of heat tolerance at the individual level. Although behavior has historically been challenging to record, technological improvements and innovations open up new possibilities to record behavior on many animals at the same time in an automated manner.

During data collection for another project (van der Sluis et al. 2020), broilers were inadvertently exposed to high barn temperatures, due to a local heat wave in the Netherlands. The project collected individual broiler location data continuously, from hatching until slaughter age, using a radio frequency identification (**RFID**) system in a small pen. This data provided information on the birds’ distribution in the pen and their general locomotor activity levels. In this study, we used those data to assess whether broiler distribution (group-level indicator) and activity levels (individual-level indicator) change in response to increased ambient temperatures. Even though the data was not collected for the purpose of the current study, the insights gained can provide clues for future research.

## MATERIALS AND METHODS

### Ethical Statement

We used data that was collected for another research project conducted at a broiler farm in the Netherlands (van der Sluis et al. 2020). The birds were not exposed to different ambient temperatures by design, but the temperature inside the barn changed over time as a result of changes in the outside temperature. The original study was considered not to be an animal experiment under the Law on Animal Experiments, as confirmed by the local Animal Welfare Body (20th of June, 2018, Lelystad, the Netherlands).

### Data Collection

This study used data collected from crossbred broilers. The data on broiler activity was collected in 2 rounds. At the start of each round, there were 40 broilers in the pen. Some of these animals were removed from the data due to early mortality (n = 1, in R1) or technical issues such as lost tags. As a result, the data from the first round (R1**;** in March 2019) included 34 male broilers, while the data from the second round (R2**;** in July 2019) included 39 broilers (all female except one due to a sexing error). The broilers were sampled from a large number of full-sib families, so they were likely unrelated. They were housed in a single rectangular pen with a size of approximately 1.8×2.6 m (i.e., 4.7 m^2^; approx. 8.5 birds per m^2^). Data were collected from 1 to 35 d of age in R1, and from 1 to 28 d of age in R2 as data collection for the original study was stopped early due to low activity resulting from a heat wave. Broilers were weighed individually every week. Feed and water were provided ad libitum. Broilers were kept under a commercial lighting and temperature schedule, with five light or dark periods within a 24-h d: dark period 1 (**D1**) from 00:00 to 03:00, light period 1 (**L1**) from 03:00 to 05:00, dark period 2 (**D2**) from 05:00 to 07:00, light period 2 (**L2**) from 07:00 to 23:00 and dark period 3 (**D3**) from 23:00 to 00:00. On day 28 of R2, the lights were on until 23:30 instead of 23:00, to allow the birds some more time to eat with the lights on at the end of the warm day (i.e., a management adjustment to the high temperature). The first 7 d from both rounds were excluded from the analyses, because the light schedule during this period was different from the light schedule during the rest of the round.

The location of the broilers in the pen was recorded over time using a passive RFID system (Dorset Identification B.V., Aalten, the Netherlands). The system and its validation are described in van der Sluis et al. (2020). Periods during which not all birds were in the pen (e.g., during weighing) were excluded from the data.

### Temperature Data

Temperature inside the barn was registered every 10 min throughout the data collection period, using a single temperature sensor placed in the middle of the barn. We determined the difference ($t_{\mathrm{diff}}$) between the realized inside temperature and the desired maximum inside temperature, because temperature requirements of broilers change as they age, and we were interested in the effects of high inside temperatures on group-level dispersion and individual-level activity. During the data collection of R2, the Royal Netherlands Meteorological Institute registered a heat wave in the area (22–26 July 2019; KNMI, 2024) and as a result the birds were inadvertently exposed to high ambient temperatures on d 25 to 28 of R2, with a maximum $t_{diff}$ of 14.6°C. It is well known that thermal comfort in animals is a function of both temperature and humidity (Bianca 1962). The temperature-humidity index (**THI**) may therefore be a more suitable metric to include as a predictor for heat stress response. Unfortunately, data on humidity inside the barn were not available, so we were not able to include THI in our analysis.

### Group-Level Dispersion

Based on the location of the birds in the pen, we calculated the distribution index (**DIS**; Equation 1, van den Oever et al. (2021)) to measure the dispersion of animals in the pen at each 15 min interval.

(1) $$DIS=\frac{\sum_{i=1}^{n}\left|N_{i}-\frac{T}{n}\right|}{2\left(T-\frac{T}{n}\right)},$$

where n is the number of antennas of the RFID tracking system, $N_{i}$ is the number of animals registered at antenna i, and T is the total number of birds. A distribution index of 0 means that the birds are spread equally over all RFID antennas, while a value of 1 means that all birds are registered at the same RFID antenna. The data used for analyzing DIS included a total of 4,700 observations, and the distribution of DIS was roughly normal.

### Individual-Level Activity

For individual-level activity, we determined the total distance moved per hour (**DMH**) in meters for each bird and each hour. These calculations are described in van der Sluis et al. (2020). The data used for analyzing DMH included 73 animals (34 in R1 and 39 in R2), and 34,241 observations (18,995 in R1 and 15,246 in R2). Most data was available for L2, because L2 was the longest time period. The distribution of DMH was roughly normal after log-transformation. Overall, DMH was lower during the dark periods than during the light periods, while the ranges of DMH values in R1 and R2 were very similar.

### Statistical Analyses

For statistical analyses, we used R (version 4.1.3) and the package lme4 (version 1.1.28). For both group-level dispersion and individual-level activity, we tested several models that differed in the interaction terms that were included. The interaction terms were between period (i.e., the different light and dark periods) and age, and between period and $t_{diff}$. Here, we only present the models (one for group-level dispersion and one for individual-level activity) that provided the best fit to the data, based on Akaike's Information Criterion (**AIC**) and visual inspection of residual plots.

### Group-Level Dispersion

To examine the effect of $t_{diff}$ on group-level dispersion, we used the model

(2) $$y_{ij}=\mu +R_{i}+P_{j}+\beta_{1_{j}}age*P_{j}+\beta_{2_{j}}age^{2}*P_{j}+\beta_{3}t_{diff}+\beta_{4}t_{diff}^{2}+e_{ij}$$

where $y_{ij}$ is the value of DIS, μ is the intercept, $R_{i}$ is the fixed effect for round (*i* = R1 or R2), $P_{j}$ is the fixed effect for light and dark periods (*j* = L1, L2, D1, D2 or D3), age is the age of the animals (8–35 days), $t_{diff}$ is the difference between the realized and aimed-for temperature, $\beta_{1_{j}}$ is the regression coefficient for age in period j, $\beta_{2_{j}}$ is the regression coefficient for squared age in period j, $\beta_{3}$ is the regression coefficient for $t_{diff}$, $\beta_{4}$ is the regression coefficient for squared $t_{diff}$, and $e_{ij}$ is the residual where $e_{ijk}\;∼\;N\left(0,\;\sigma_{e}^{2}\right)$.

### Individual-Level Activity

DMH was first examined using descriptive statistics and histograms. DMH was considerably right-skewed, and we therefore decided to transform the data using a natural logarithm. To examine the effect of $t_{diff}$ on activity, we used the linear mixed model

(3) $$y_{ijk}=\mu +R_{i}+\;P_{j}+\beta_{1}age+\beta_{2}t_{diff}+\beta_{3_{j}}age*P_{j}+\beta_{4_{j}}t_{diff}*P_{j}+Animal_{k}+e_{ijk},$$

where $y_{ijk}$ is the log-transformed value of DMH, $\beta_{1}$ is the regression coefficient for age, $\beta_{2}$ is the regression coefficient for $t_{diff}$, $\beta_{3_{j}}$ is the regression coefficient for age in period j, $\beta_{4_{j}}$ is the regression coefficient for $t_{diff}$ in period j, $Animal_{k}$ is the random effect of the individual where $Animal_{k}\;∼\;N\left(0,\sigma_{A}^{2}\right)$, $e_{ijk}$ is the residual where $e_{ijk}\;∼\;N(0,\;\sigma_{e}^{2}$), and all other terms are the same as in Equation 2. We did not observe strong deviations from normality or heteroscedasticity from visual inspection of the model residuals. For all analyses, the level of statistical significance was set at 0.05.

## RESULTS AND DISCUSSION

### Group-Level Dispersion

**Figure 1:**
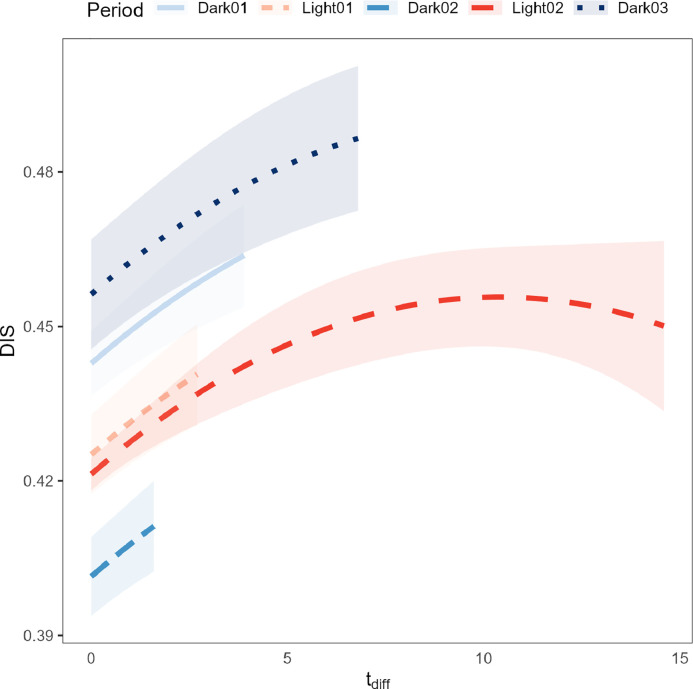
Model predictions of the distribution index (DIS, y-axis) as a function of $t_{\mathrm{diff}}$ (x-axis) and period during the day (differently colored lines). Lines are truncated at the highest observed $t_{\mathrm{diff}}$ within each period. Shaded areas represent the 95% confidence interval of the model predictions.

All factors included in the model had a significant effect on DIS, including the interaction between age and period, and age^2^ and period. For all periods, DIS decreased almost linearly when age increased from 8 to 20 d, but slightly increased again when age increased from 20 to 35 d, especially in the light periods (results not shown). A potentially confounding factor here is that as the birds grew, less of them fit together in a small area (i.e., on one RFID antenna), thus lowering the maximum potential DIS value over time. DIS increased when $t_{diff}$ increased from 0 to about 9°C, and slightly decreased when $t_{diff}$ increased from 9 to 14°C in L2 (Figure 1). This result suggests that animals tend to move closer together as the temperature in the barn increases. This result seems counterintuitive, as one might expect birds to move farther apart to allow more airflow around the body for heat dissipation. Unfortunately, we were not able to study what caused this clustering behavior because video recordings or data on specific behaviors were not available.

Indeed, our results are not in line with findings from earlier studies. Pereira et al. (2020) used video data to track birds over a short period of time, and computed a cluster index to assess clustering behavior under ambient temperatures of 26, 29, and 35°C. Their results showed that animals tended to cluster less with higher ambient temperatures. It must be noted that their study setup was different from the current study, which might explain some of the discrepancy between these results: 1) they used data from a single period of only 7 min while we used data at 15 min intervals during the entire growing period, 2) they used a different clustering calculation (a so-called cluster index, which can be calculated from area and perimeter size of segments in processed images), and 3) their study was on laying hens instead of broilers. Nonetheless, alternative hypotheses that might explain our observations include that birds prefer to settle in an area where the air or floor is slightly cooler than in the rest of the pen, for example, due to a draft, or that birds tend to eat or drink at the same time more often, for example, during parts of the day when it is cooler (Fernandes et al., 2021). Further research is required to test these hypotheses.

Our results also showed that when barn temperature reached about 10°C above the desired maximum temperature, birds tended to move further apart as the temperature increased. It is important to note, however, that there were a limited number of observations at such high temperatures, and these observations were all from period L2. These results should therefore be interpreted with caution. Nevertheless, we hypothesize that with extremely high temperatures, the advantage of being located in a cooler place in the pen may not outweigh the disadvantage of being close to conspecifics. At very high temperatures, birds may prefer to increase their distance to conspecifics so that it becomes easier to dissipate body heat.

### Individual-Level Activity

**Figure 2:**
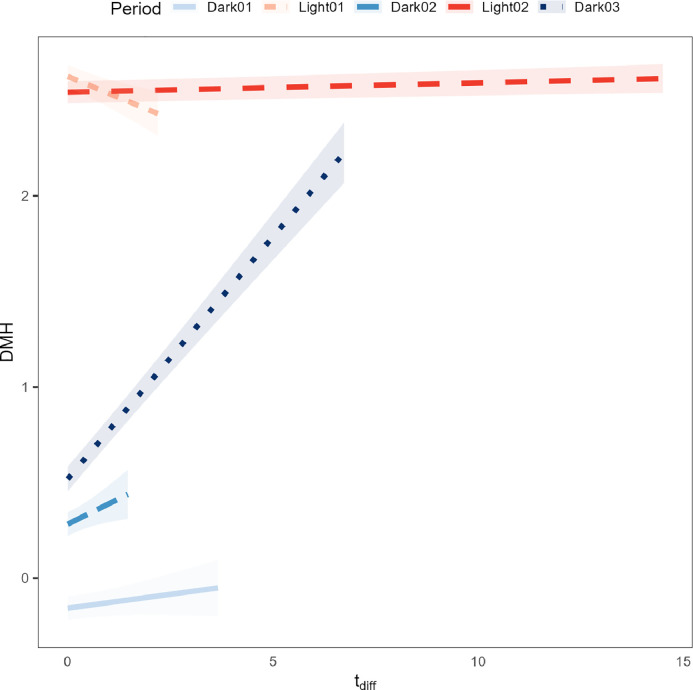
Model predictions of distance moved per hour (DMH, y-axis) as a function of $t_{\mathrm{diff}}$ (x-axis) and period during the day (differently colored lines). Lines are truncated at the highest observed $t_{\mathrm{diff}}$ within each period. Shaded areas represent the 95% confidence interval of the model predictions.

The results from the linear mixed model on DMH showed that there was a significant interaction effect between period and age. As expected, DMH was higher in the light periods than in the dark periods, and decreased with increasing age for all periods, except for D1, where the effect of age was negligible (results not shown). Furthermore, there was a significant interaction effect between period and $t_{diff}$ on DMH. With increasing $t_{diff}$, DMH decreased in L1 (Figure 2), which is in accordance with results from earlier studies (Schiassi and Silva 2015; Branco et al. 2020; Fernandes et al., 2021). However, DMH remained stable with increasing $t_{diff}$ in D1, L2, and D2, and strongly increased in D3.

The difference in the effect of $t_{diff}$ on activity between light and dark periods may be caused by several factors. First, birds may display compensatory behavior: during the hottest part of the day, birds may eat less (Rodrigues et al., 2019), and they need to compensate for this when the temperature drops in subsequent periods. This compensatory behavior is reflected in the shift of activity from the light to the dark periods in the current study. Second, after a period of extreme heat (e.g. during L2), birds may need to dissipate their body heat. Possibly, they increase their activity to increase the airflow around their body, which may explain why activity increases in D3. Third, exposure to heat stress may disrupt the birds’ general activity pattern. Typically, birds are active during the light periods and inactive during the dark periods. With high ambient temperatures, the resulting stress response may disrupt their normal activity pattern.

There was no effect of $t_{diff}$ on DMH during the hottest and longest part of the day (L2). This was surprising, because we expected that birds would move less with higher temperatures to reduce heat production. A possible explanation for this result is that although the birds are prone to move less, they are also motivated to drink more (Li et al. 2015; Rodrigues et al., 2019; Kim et al. 2022; Cartoni Mancinelli et al., 2023). As a result, they may move to and away from the drinkers more often. In addition, birds may become more restless when the temperature is well above their thermoneutral zone, which would increase their activity (Li et al. 2015). Unfortunately, we could not confirm these hypotheses based on the data that were available. Future research including visual or camera observations could be of great added value to assess what behaviors constitute the majority of the activity under different ambient temperatures.

A complicating factor in our data is that the heat wave coincided with the last week of the production data, and therefore age and temperature effects are somewhat confounded. As generally observed for broilers, activity levels decreased with age in this study. Examination of broiler activity recorded with high temperatures occurring earlier in life could help disentangle the effects of age and temperature.

The model included a random animal effect to account for differences in mean activity between individuals, which explained 8.4% of the phenotypic variation. This suggests that there is some variation between animals in their activity levels, which is partly heritable (Ellen et al., 2024 in preparation). The findings that activity levels vary between individuals and that activity is affected by temperature suggest that activity could be an interesting indicator trait for heat tolerance in broiler chickens. This suitability should be confirmed with further research that includes data on production traits from a larger number of animals that were exposed to different barn temperatures.

### Future Outlook

Overall, the results of this study show that barn temperature affects both group-level dispersion and individual-level activity in broilers. Contrary to expectation, birds clustered more in response to high barn temperatures. In addition, the effect of high barn temperatures on individual activity differed between periods of the day. Most notably, activity increased at the end of the day with higher barn temperatures, which likely coincides with the barn cooling down. Together, these results can help to design early-warning systems for heat stress in broilers, as well as potential automated detection of cold or hot spots in the barn, based on behavioral indicators. It should be noted, however, that such a system probably requires data on behaviors that are more specific to heat stress than dispersion and general activity level alone (e.g., drinking and panting behavior), given that many factors can (simultaneously) affect dispersion and activity in broilers. Finally, the results suggest that activity data at the individual-level can potentially inform about differences in heat tolerance between broilers, which could be interesting for breeding purposes.

## DISCLOSURES

The authors declare no conflicts of interest.
